# Biomimetic Molecular Signaling using DNA Walkers on Microparticles

**DOI:** 10.1038/s41598-017-04316-1

**Published:** 2017-06-22

**Authors:** Tulsi Ram Damase, Adam Spencer, Bamidele Samuel, Peter B. Allen

**Affiliations:** University of Idaho, Dept. of Chemistry, 001 Renfrew Hall, 875 Perimeter Dr, Moscow, ID 83844-2343 USA

## Abstract

We report the release of catalytic DNA walkers from hydrogel microparticles and the detection of those walkers by substrate-coated microparticles. This might be considered a synthetic biology analog of molecular signal release and reception. One type of particles was coated with components of a DNA one-step strand displacement (OSD) reaction to release the walker. A second type of particle was coated with substrate (or “track”) for the molecular walker. We distinguish these particle types using fluorescence barcoding: we synthesized and distinguished multiple particle types with multicolor fluorescence microscopy and automated image analysis software. This represents a step toward amplified, multiplex, and microscopically localized detection based on DNA nanotechnology.

## Introduction

DNA nanotechnology has demonstrated the ability to build static objects and dynamic machines at the nanometer scale with molecular precision. Designed DNA reaction-diffusion systems have performed feats of biomimetic computation: they have recapitulated oscillating patterns in a reaction diffusion system^1^ and are moving toward biomimetic regulatory and signaling pathways^2^. DNA origami^3^ has built 3D objects^4^ from DNA that move and react to stimuli^5^. However, these systems are not practical for observing chemical information exchange among microscale objects. We hope to move toward tools that could be used to explore biological phenomena within the cellular microenvironment such as cytokines and extracellular miRNA. These local signals are important biomarkers for nerve injury and cancer^6–8^. We present one step toward more practical bioanalytical applications of DNA nanotechnology. This paper shows a system that can release a molecular walker from one particle type and detect that walker using a second particle type.

DNA circuits are DNA-DNA reactions in which base-pairing rules are used to construct specific reactions and reaction networks. The field of DNA computation has demonstrated a remarkable array of such DNA circuits for performing logical operations and amplification. One simple and robust DNA-DNA reaction is the one step strand displacement (OSD) reaction^9^. In this reaction, an input single stranded DNA (ssDNA) oligonucleotide binds to a 2-part DNA complex. The interaction is initiated using a short single stranded “toehold.” The input ssDNA displaces an output strand and forms a longer duplex and a more stable product. The displaced ssDNA strand may then be used as an input for further such OSD reactions. Complex pathways can be designed. In one example, a set of more than 30 OSD reactions were integrated to compute the square root of a four-bit binary number^10^. Arrays of DNA-bearing particles have also been used to execute complex communication. DNA polymerase was used to drive rationally designed DNA circuits on particle surfaces^11^.

Enzyme-free DNA circuits can also amplify a molecular signal. In catalytic amplifiers, one input can produce many output molecules. One example is the catalytic hairpin assembly (CHA) reaction^12^. The CHA reaction starts with unstable, kinetically trapped DNA hairpins which are catalytically assembled into a more stable double stranded DNA (dsDNA) duplex. Many detection methods can report the amplified output^13^. Enzyme-free reactions using DNA circuits offer some advantages over enzymatic amplification: they can be dehydrated, are robust to temperature change and are more easily re-designed to deal with new targets and circumstances^14^. Our work has shown that these reactions can also be immobilized on particles; the output of the reaction can then be measured with fluorescence microscopy^15^.

Microparticle-based sensing offers advantages in terms of multiplexing^16^, simplicity^17^ and automation^18^. Other labs have demonstrated particle multiplexing using lithographically generated shape^19^, size^20^ and fluorescence spectrum^16^. Microparticles have been used for affinity capture as well as for sandwich assays read out by flow cytometry or microscopy. The use of a fluorogenic assay simplifies the experiment further as it alleviates the need to wash the particles^20^.

We set out to immobilize a DNA circuit amplifier on the surfaces of hydrogel microparticles. We used a molecular walker based on the Zhang lab’s entropically driven amplifier (EDA)^21^. The EDA is designed so that two input molecules react to generate three product molecules. The resulting gain in entropy drives the reaction. One of the reaction products is a regenerated catalytic domain. (see Supplemental Information Fig. SI1 for a detailed circuit comparison between our design and that by Zhang *et al*.^21^). By incorporating two catalytic domains on the same molecule (a divalent DNA strand displacement catalyst), we bias the molecule to remain persistently on a single particle bearing the substrate. Previous work has shown that this basic approach produces catalytic activity that is constrained to the surface of a particle^22^. We refer to this divalent catalyst as a DNA walker. It takes a “step” when one catalytic domain completes a reaction and is released, but the other domain remains bound to substrate. As long as one leg re-binds before the other leg is released, the walker will crawl over the surface of the particle, leaving behind a trail of fluorescent reaction product.

We made particles that released walker molecules and other particles that detected the walkers. We use fluorescence barcoding to identify the two particle types. We released a DNA walker from blue “release microparticles.” We detected this DNA walker with green “detector microparticles.” Different particle types were generated with barcoded fluorescence intensity ratios in the green (λ_ex_ 490 nm) and blue (λ_ex_ 400 nm) channels in order to identify the specific particle type in a multiplex analysis. The detector particles were generated with the EDA substrate (TR-SB-Q_EDA_) immobilized on their surface. The molecular walker persisted on the surface of the detector particles and generated significant fluorescence despite extremely low average concentration. In our design, a fluorogenic DNA reaction product is detected in the red channel (λ_ex_ 580 nm). We demonstrate that the multiplex capability can be extended further. We generated three particle types and used custom automated image analysis to selectively quantify the red fluorescence response among blue green and teal particles.

## Results

### Generation of hydrogel particles

We synthesized fluorescent hydrogel microparticles coated with DNA. We generated these polyacrylamide hydrogel particles by emulsion and radical polymerization. We synthesized our own particles rather than using commercially available particles because commercial fluorescent particles are too bright. Even a small amount of bleed-through into the red channel distorts the quantification.

Acrylamide/bis-acrylamide prepolymer was dispersed into microdroplets by extrusion or homogenization in mineral oil. The microdroplets were polymerized into microparticles. DNA was included in the prepolymer mixture. This DNA complex was synthesized with a cholesterol and an acrydite modification. The hydrophobic cholesterol caused the DNA to associate with the aqueous/oil interface when the water was dispersed in the oil. When the hydrogel polymerized, it incorporated the acrydite into the polymer. This locked the DNA in place at the surface of the particle (see Methods). In our previous work^15^, we compared the cases with and without the cholesterol-modified DNA. Fluorescence microscopy showed localization of the fluorescent DNA to the margin of the particle only in the case where the cholesterol was added. We applied Texas Red modified DNA complex (TR-Q_OSD_ or TR-SB-Q_EDA_). We used an OSD reaction to remove the cholesterol-DNA and add the TR complex.

### Microscopically localized detection of diffusing DNA walker

**Figure 1:**
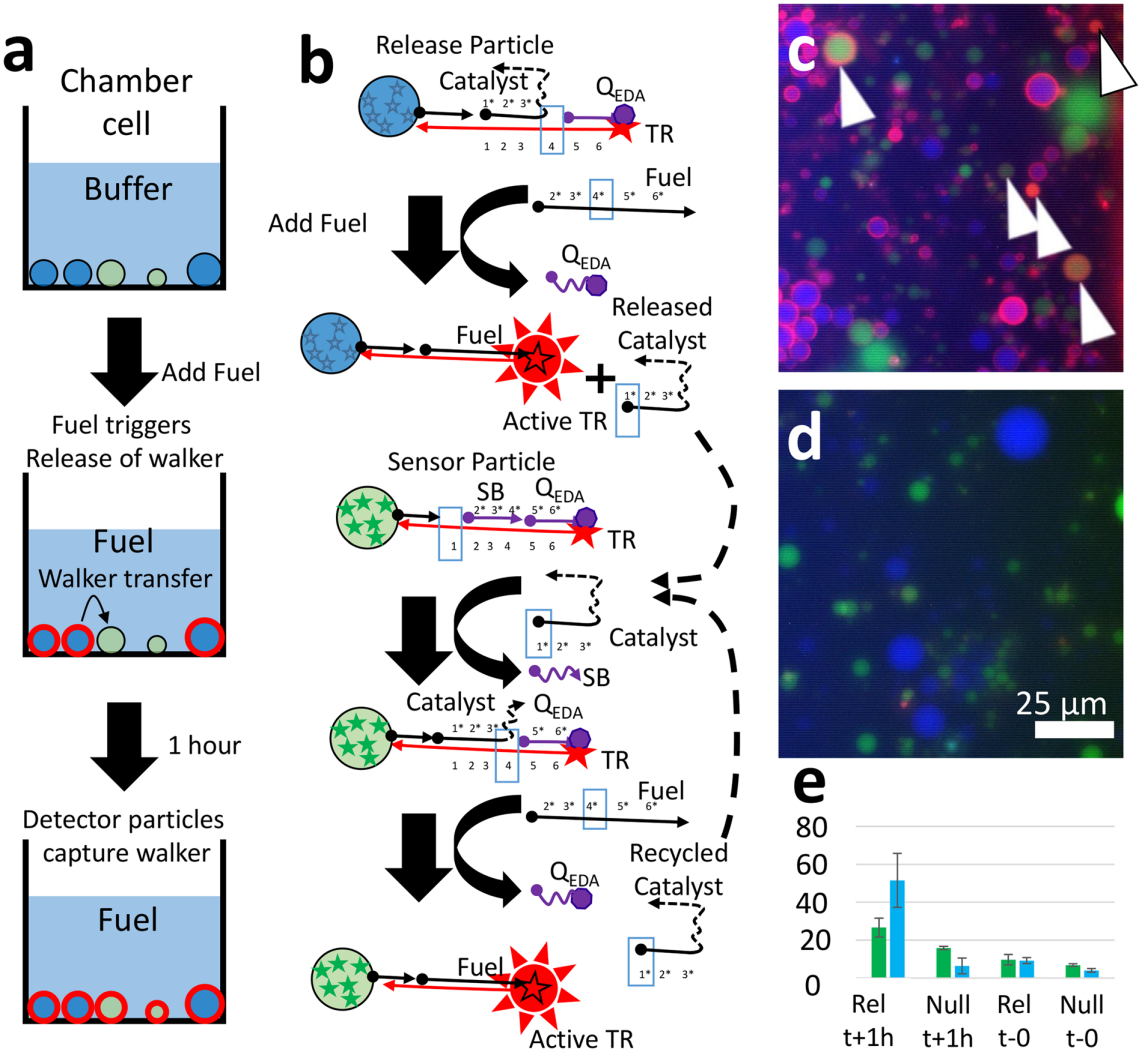
Demonstration of molecular walker release and capture. (**a**) A schematic shows the experimental steps for walker release and detection (**b**) A schematic shows how Cascade Blue dyed (blue) particles released the DNA walker in response to fuel; the DNA walker diffused to fluorescein-dyed (green) sensor particles and acted to de-quench Texas Red on their surfaces (arrows). (**c**) Fluorescence micrograph shows fluorescein dyed sensor particles and cascade blue release particles after incubation for 1 hour with fuel. (**d**) Fluorescence micrographs of the control system with blue particles bearing SB instead of the DNA walker after incubation for 1 hour with fuel. (**e**) Bar graph shows the average red fluorescence of the blue and green particles before and after addition of fuel from 8 or more images. Error bars represent the standard deviation of the particle intensities.

We released DNA walker molecules from release particles and detected these walkers using a set of detector particles. The detector particles were created with an immobilized substrate for the entropy driven amplifier (EDA) circuit on their surfaces. The mechanism of the EDA is shown in Fig. 1(b). This circuit was adapted from the EDA published by Zhang *et al*.^21^. The functionality of the circuit in free solution was also verified by gel electrophoresis (see Fig. SI1). The walkers were a divalent version of the EDA catalyst.

Release and detector particles were settled on the surface of a chamber slide and viewed with a fluorescence microscope. The walkers were released from blue-dyed particles. Green-dyed detector particles captured and reported the walkers. The walker catalyzed a fluorogenic EDA reaction, causing the green particles to display red fluorescence. Figure 1(c) shows representative initial and final fluorescence state of the release (blue) and detector (green) particles. Initially, no particles were fluorescent in the red channel. A single-stranded DNA molecule was added to the system (denoted “fuel”). This fuel molecule served two purposes: 1. it released the molecular walkers from the blue-dyed particles by an OSD reaction; 2. it participated in the catalytic reaction of the molecular walker on the surface of the green particles.

The fluorescent rings around some particles correspond to many fluorophores within the focal plane of the microscope. Because our particles have a wide size distribution, some particles are not in focus. These do not show clear rings of fluorescence and sometimes appear as a solid spot. Other particles may contain fewer DNA molecules. We compensate for this by averaging data from many particles using an automated image analysis system as described below.

We collected 8–14 images before and after adding the fuel molecule. We processed these images using automated image analysis software to extract average responses from the blue particles and green particles. The results show that the blue particles become highly fluorescent after adding fuel. The green particles also become fluorescent after adding fuel; this only occurs when DNA walker is present and so we can infer that the DNA walker was transferred to the green particles. The negative control case (Fig. 1(d)) shows that when the release particles lack the DNA walker (blue particles are coated with TR-SB-Q_osd_ instead), neither the blue nor the green particles show strong red fluorescence. Grayscale fluorescence intensity images for the components of the false-colored micrographs are included in Supplemental Information as Fig. SI2.

**Figure 2:**
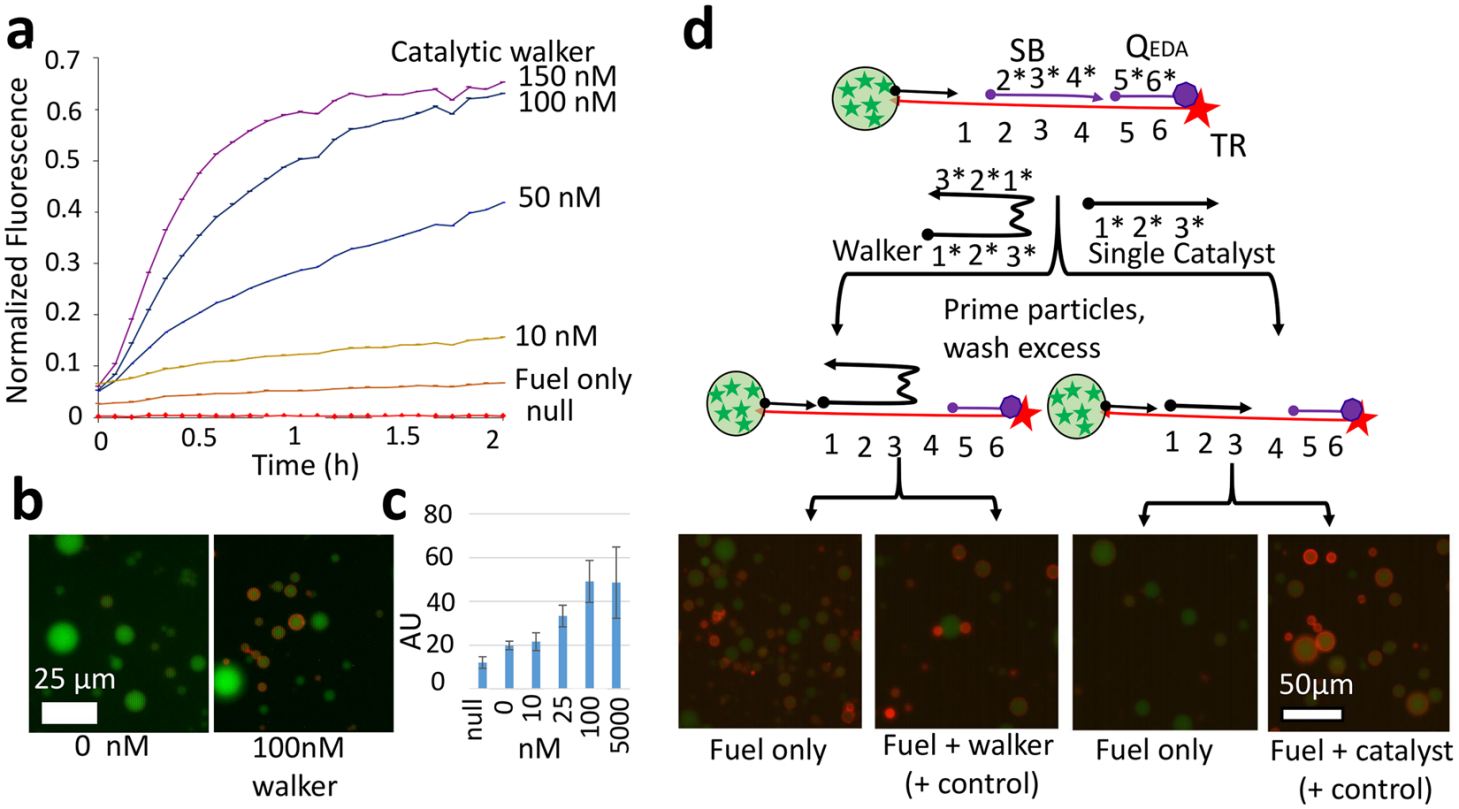
Demonstration of the catalytic circuit in solution. (**a**) Graph of fluorescence over time shows how the fluorescence of the DNA increases at different DNA walker concentrations. (**b**) End-point fluorescence micrographs of detector particles after incubation with DNA walker for one hour. (**c**) Average particle fluorescence as observed in the fluorescence microscope (error bars are the standard deviation of the averages of 3 images) (**d**) Schematic and end-point fluorescence micrographs showing the high persistence of the walker.

As shown in the schematic in Fig. 1(a), 1 μl of dilute particles were added to 40 μl of reaction buffer in a chamber slide on the microscope. At most, the particles carried 1 pMol of immobilized catalyst (assuming zero losses during particle synthesis, washing, and dilution). This gives a maximal bound of 2.5 nM final average concentration. This is already lower than the LOD for bulk concentrations of catalyst (as described below in Fig. 2). In fact, most (>90%) of the particles were removed by the subsequent washing (to leave only a scattering of particles adhered to the surface). A more reasonable estimate of the average concentration is less than 0.3 nM. Therefore, we conclude that the transient, high concentration after release produced the response on the green particles.

### Mechanism and performance of DNA walker

The green particles detect the walker only because the walker is persistently associated with the particle surface. Our detector particles responded to catalytic walker at a concentration that was transiently high (immediately after release) but which decreased to sub-nanomolar concentrations after diffusion. The detector particles are not sensitive enough to detect the walker at these low concentrations. We tested the limit of detection of the DNA walker in order to interpret the release-and-capture experiment.

We first tested the limit of detection for the detector circuit in solution (without particles) against DNA walker in solution. The DNA walker permits the rearrangement of the other DNA species into a more stable conformation. In our designed reaction, the product is fluorescent. The rate of rearrangement into the fluorescent product configuration is very slow without the DNA walker; the reaction rate increases by several orders of magnitude in the presence of the DNA walker.

We adapted a catalytic circuit originally published by David Zhang *et al*.^21^ Our circuit design is shown in Fig. 1(b). We made three changes to the original design: (1) we simplified the circuit to include the fluorogenic reporter in the substrate; (2) we reoriented the design to allow for the key chemical modifications in the appropriate positions (e.g. the acrydite is only available as a 5′ modification); (3) our version of the catalyst was divalent with two catalytic domains. This divalent catalyst is called a DNA walker because it remains persistently bound to the particles over multiple turnovers. The use of polyvalency to allow for walking behavior has been presented elsewhere^12, 22, 23^. The release-and-catch experiment shown in Fig. 1 highlights the best performance of the system. For a detailed comparison of our circuit to the original, see Supplemental Information, Fig. SI1. Figure 2 shows the operation of this circuit and that it was immobilized on the surface of the microparticles. The particles become fluorescent when the DNA walker was added.

Individually, fuel or DNA walker are unable to displace the quencher from the Texas Red complex. Once the DNA walker displaces the SB (blocker) strand, the fuel can displace the quencher and DNA walker (so that the catalytic domain is recycled). We combined substrate (TR-SB-Q_EDA_) and fuel in a 384 well plate with various concentrations of the DNA walker. The resulting increase in fluorescence is shown in Fig. 2(a). The plate reader data (solution phase) suggests that we are able to detect a presence of 10 nM initiator.

We performed the catalytic EDA reaction with TR-SB-Q_EDA_ on the surface of detector particles. We allowed a range of DNA walker concentrations to react with the particles for 1 hour. We examined the results using fluorescence microscopy. Fluorescence micrographs are shown in Fig. 2(b). The data show a clear increase in the endpoint fluorescence as the quantity of DNA walker increases. We analyzed image data from six conditions. We measured the average particle fluorescence over all of the particles in a given image. The results are shown in Fig. 2(c). Microscopy data (on particles) suggest a limit of detection of 16 nM with the LumaView LED-based microscope. The LOD in terms of the average solution phase walker concentration is not adequate to detect the ~0.3 nM average concentration of walker in the release-and-catch experiment. The transiently high concentration after release accounts for the detection of walker by the detector particles.

### Divalent walker is necessary to detect transient exposure

We performed controls to show that the double catalyst is a persistent walker on the surfaces of microparticles. We also show that the walker (with two catalytic domains) is necessary for high persistence and strong response to transient exposure to catalyst. A non-walker catalyst (with only a single catalytic domain) has lower persistence on the particle surface. We transiently exposed substrate particles to 25 nM double catalyst or 50 nM single catalyst (equivalent number of catalytic domains). After one minute, the unbound catalyst was removed by centrifugation and resuspension in buffer. Even the single catalyst binds to the substrate with 22 bases of hybridization. This has a binding energy of 27 kcal/mol and the dissociation constant of 1 × 10^−20^ (per OligoCalc^24^). This is essentially a permanent association (without fuel to provide the energy to displace the catalyst). The double catalyst interacts even more strongly. The resulting “primed” particles were then incubated with fuel for one hour (See Fig. 2(d)). Only the double catalyst had significant response. The persistence of the double catalyst allowed it to ‘walk’ through multiple catalytic turnovers while the single catalyst was essentially immediately released.

### Mechanism and performance of the OSD reaction

**Figure 3:**
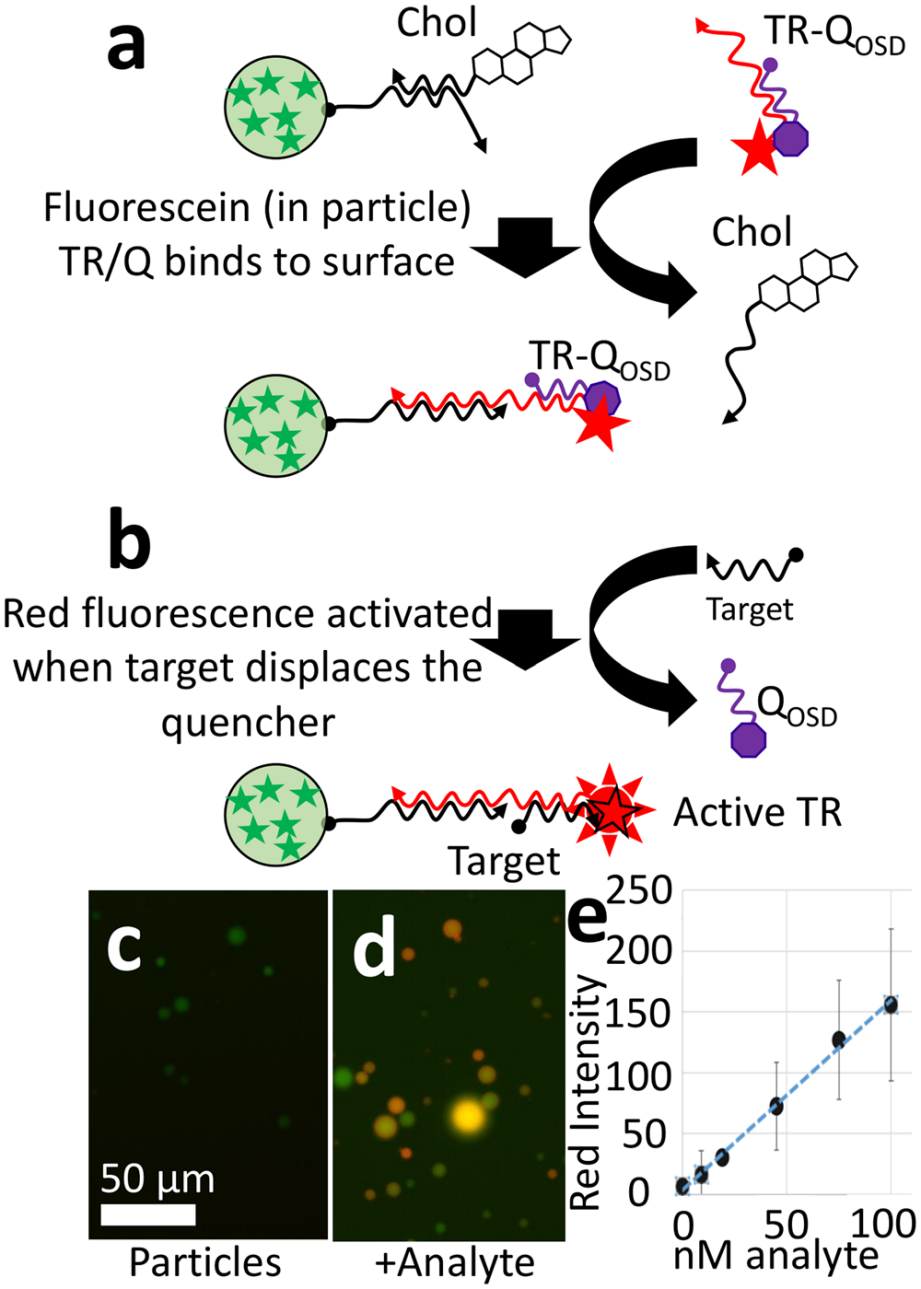
Demonstration of OSD reaction on particle surface. (**a**) Schematic shows how sensor particles are prepared. Cholesterol DNA is displaced by the quenched detector complex (TR-Q_OSD_). (**b**) A schematic shows how the presence of TR-Q_OSD_ was verified. A specific ssDNA displaces the quencher molecule (Q_OSD_). This results in increased Texas Red fluorescence on the microparticle surface. (**c**) Fluorescence micrograph shows initial green fluorescence of the sensor microparticles. (**d**) Fluorescence micrograph shows the increased red fluorescence after addition of ssDNA. (**e**) Calibration curve shows the mean fluorescence and standard deviation among several images at various concentrations of ssDNA.

To show the advantages of amplification with the walker, we also characterized the sensitivity of a simple strand displacement reaction. The sensitivity of the one-step strand displacement (OSD) reaction was lower than that achieved by amplification with the DNA walker. This characterization also demonstrates the reactions used to build and operate our detector particles. OSD reactions were used to immobilize the molecular walker substrate complex on the particles and to release the molecular walker from the release particles. OSD reactions are not catalytic but are simple and reliable. We demonstrate the functionality of the OSD reaction in Fig. 3. A simple fluorogenic complex on particles is activated using an OSD reaction. Particles were generated containing fluorescein-modified poly-T (without cholesterol, to visualize the particles in green fluorescence prior to surface detection). Figure 3(a) shows how green microparticles were coated with the fluorogenic DNA complex and how the cholesterol-modified strand was removed. The particle is initially coated with the acrydite and cholesterol DNA. When this particle encounters its complementary ssDNA, it hybridizes to the surface-bound complex and displaces the cholesterol DNA.

A second OSD reaction can also remove the quencher (see Fig. 3(b)). The displacement of the quencher results in the activation of the fluorophore on the microparticle surface. This shows that the TR complex was added and is available to react further. Figure 3(c) shows the initial green fluorescence of the particles; Fig. 3(d) shows the results after the ssDNA displacer was added to the mixture. The particles show a clear increase in red fluorescence. The red fluorescence intensity was quantified using automated image analysis software (see below).

The calibration curve in Fig. 3(e) shows the average intensity of the particles in 4–6 images. The standard deviation from image to image is shown as the error bars. The results indicate that we can achieve a limit of detection (LOD) of approximately 25 nM with the OSD reaction and the Lumaview LED microscope. Using a more sophisticated and sensitive confocal microscope, the limit of detection was <10 nM for a ssDNA displacer (as shown in Supplemental Information, Fig. SI3).

### Blue/green fluorescence barcoding

Our image analysis software required a clear distinguishing feature between release and detector particles. We used the fluorescence response in green and blue to identify the two particle types. We generated particles with very different fluorescence emission profiles. We performed controls and characterizations to show that we could detect specific reactions on barcoded particles using OSD reactions. We generated three different types of particles containing different ratios of green and blue fluorophores. By immobilizing different fluorogenic sensors on the surfaces of each type of particle, we were able to detect three different DNA species.

**Figure 4:**
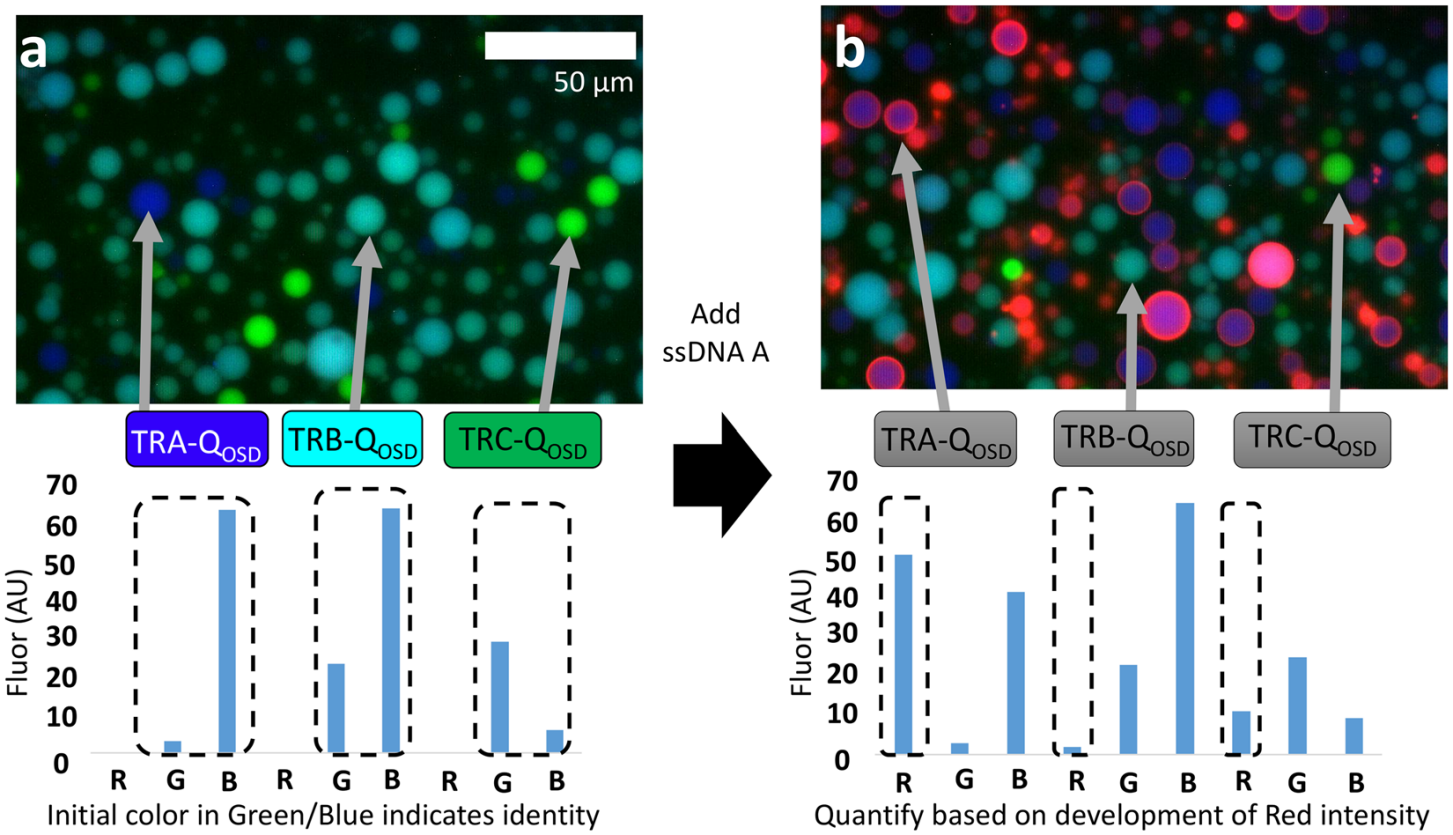
Multiplex particles using blue and green fluorescence. (**a**) Fluorescence micrograph and bar graph show the microparticles and corresponding fluorescence intensities in the red, green and blue channels before addition of ssDNA A. (**b**) Fluorescence micrograph and bar graph show resulting fluorescence intensities of the microparticles after addition of ssDNA A.

Each immobilized DNA complex bears a quenched Texas Red fluorophore and a toehold sequence specific for one ssDNA displacer. As a consequence, the three particle types display red fluorescence in response to the addition of a particular ssDNA. Figure 4 shows fluorescence micrographs before and after adding ssDNA A which is specifically reported by TR_A_-Q_OSD_. The OSD reaction proceeded as outlined above (and shown in Fig. 4(a)). Green, blue, and teal particle types can be identified by the specific ratio of red and green fluorescence. The concentration of ssDNA A could be inferred by the red fluorescence immobilized on the blue particle type (Fig. 4(b)). Grayscale fluorescence intensity images for the components of the false-colored micrographs are included in Supplemental Information as Fig. SI4.

This simple case shows that the particles respond specifically despite the fact that the sequences of the three ssDNA displacers are the same over two thirds of their length. Strand displacement probes of this kind in solution have high specificity^25^. Our particle based application of this system shows a strong signal in the red channel. The use of fluorescein, pacific blue and Texas Red dyes at these low concentrations did not produce bleed through of the blue and green fluorescence into the red channel (which would complicate quantification).

### Multiplex fluorescent data analysis with Python

**Figure 5:**
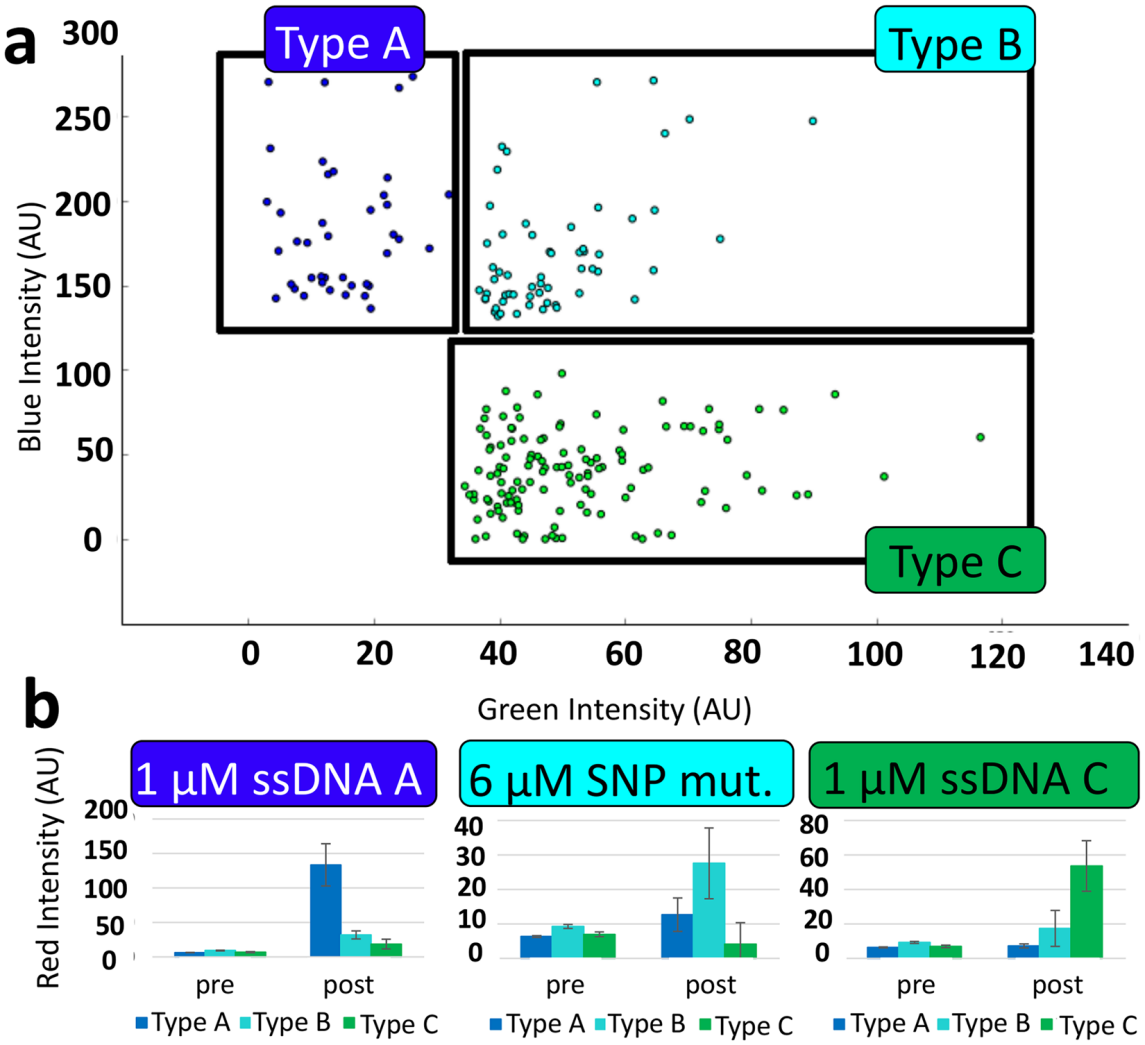
Results of automated image analysis using Python. (**a**) Images of particles are sorted according to their blue/green fluorescence. A scatter plot shows how the blue, green and teal particle data are sorted. (**b**) Red fluorescence intensity is graphed before and after adding the respective ssDNA. Error bars are the standard deviation of the mean of 9 images.

To process many images of release and detector particles, we used automated image analysis software (see Supplemental Information). We show that this software can discriminate three different particle types and quantify their red fluorescence. Blue, teal, and green particles were generated as above to respond to ssDNA A, B, and C, respectively. Experimental samples of a mixture of all three particle types were exposed to high concentrations of ssDNA A, and C. We also exposed the particle mixture to a single nucleotide polymorphism (SNP) mutant of ssDNA B. This was designed to differ by one base from ssDNA B. The resulting fluorescence micrographs were processed using our software. That program examines the images and finds bright objects in the blue and green channels. The program sorted the objects according to their green and blue fluorescence as shown in the scatter plot in Fig. 5(a). For example, objects showing strong blue fluorescence and low green fluorescence were designated as Type A particles. Teal and green objects were processed similarly.

Type A and C particles responded specifically to their respective ssDNA. No particles responded strongly to the ssDNA B SNP mutant. Type B particles responded at less than 50% maximum intensity even at extremely high concentration of 6 μM. The highest crosstalk was between the ssDNA B SNP and the blue (Type A) particles. This may be due to the two bases of sequence similarity between ssDNA A and B. Based on these results, we chose the Type C acrydite DNA to attach the TR complex for the release particles and detection particles (the TR complexes in Fig. 1).

The software was able to discriminate the response of the three particle types to three different ssDNA species. Despite potential issues surrounding the OSD reaction specificity, the software reliably found and sorted particles in the image data. Red fluorescence from blue, green and teal particles was measured with no significant interference from the green or blue barcoding channels.

## Discussion

We set out to show that a DNA circuit could amplify a molecular signal exchanged between microparticles. We achieved this using a DNA walker released from blue-dyed hydrogel microparticles and captured by green-dyed hydrogel microparticles. The green-dyed detector particles were synthesized with the walker’s fluorogenic substrate on their surfaces. This was meant to be similar to the exchange of soluble chemical signals among cells.

Hydrogel particles with a similar size to cells were synthesized by dispersion polymerization. The DNA was self-organized on the particle surfaces with a cholesterol modification. The particles can be identified by a fluorescence barcode in the blue and green wavelengths with simultaneous quantification by red fluorescence. Simple OSD reactions that de-quench Texas Red fluorophores showed a limit of detection of 25 nM (limited by the sensitivity of our microscope). The DNA walker reaction can also de-quench Texas Red fluorophores. The DNA walker reaction could be detected with a LOD of 16 nM with the LED-based epifluorescence microscope. It also allowed for capture and detection of a DNA walkers released from particles on the surface of the chamber slide.

The walker enabled detection by its persistent association with the detector particle. The walker was a divalent catalyst for the EDA circuit. The immobilized OSD and non-walker EDA reactions are both insufficiently sensitive to detect the DNA released from other particles. When DNA is released, it is rapidly diluted. The total quantity of release particles was limited to the small number retained on the microscope slide (and thus, the total quantity of DNA walker was very small). We conservatively estimate that the final concentration of DNA walker in the sample was sub-nanomolar. Without some way to retain the molecules at the initial concentration, the final concentration would be far below the limits of detection. The polyvalent DNA walker was retained by the detector particles through multiple turnovers of the EDA reaction. The released DNA walkers were thus successfully reported by the detector particles because of the high transient concentration upon release.

This work shows the utility of a particle platform to report the activity of DNA reactions. As this technology matures, it may help to enable the detection of diffusing analytes locally around cells. This would require a “transducer” such as a conformation changing aptamer^26^ that could activate the walker upon binding.

## Methods

### Generation of hydrogel particles

**Figure 6:**
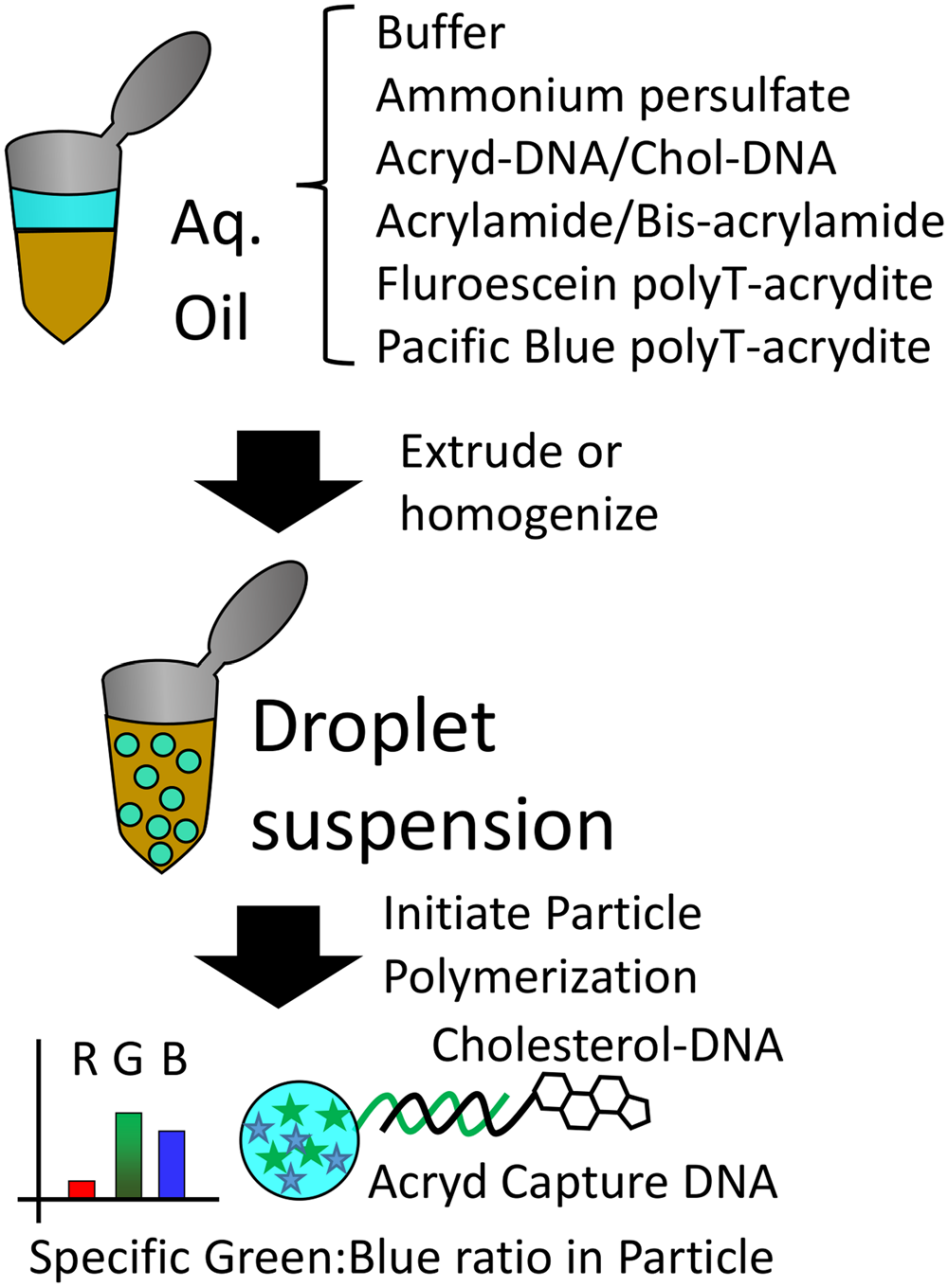
Schematic of procedure for generating DNA-decorated, polyacrylamide hydrogel microparticles.

DNA was purchased from IDT (Integrated DNA Technologies Coralville, IA) and was used without further purification unless otherwise noted. See Supplemental Information for sequence information. Our technique for generating hydrogel particles has been described in detail elsewhere^15^. Briefly, we prepared 100 μl of a prepolymer mixture containing 10 μM cholesterol/acrylamide-modified DNA complex, 20% w/v acrylamide (Research Products International Corp, IL 60056 USA), fluorescently labeled poly-T DNA (fluorescein and/or Pacific Blue conjugated acrydite-modified DNA as described below), 10% w/v ammonium persulfate (Bio-Rad Laboratories, Hercules, CA), and phosphate buffer (pH 6.8, 50 mM sodium phosphate, 50 mM sodium chloride, both from EMD Chemicals, Gibbstown, Germany). This mixture was homogenized in 1 ml a solution of 1% Span-80 (Sigma-Aldrich, St Louis) in mineral oil (Cococare Products, Dover, New Jersey) by shearing with a 2 mm steel shear mixer in a rotary tool for 4 min at 10,000 rpm. To this solution we added 8 μl of TEMED (Bio-Rad Laboratories, Hercules, CA) to initiate polymerization. The suspension was placed in a nitrogen atmosphere and shaken to remove oxygen. The suspension was allowed to polymerize for 60 minutes (see Fig. 6). The suspension was then washed free from oil by repeated centrifugation and resuspension in 70% ethanol (Pharmco-AAPER, Brookfield CT). After all oil was removed, the suspension was dried in a gentle stream of air for ~30 min. The particles were then resuspended in the working buffer. The fluorogenic DNA complex (the appropriate version of TR and Q) was annealed by heating to 85 °C for 2 min then cooling at 0.1 °C per min to room temperature in working buffer and then incubated with the particles. The TR strand was designed with a toehold and complementary region to the acrydite-modified DNA. It displaced the cholesterol-modified DNA by an OSD reaction. The particles were then washed three times with working buffer and refrigerated until use. The resulting range in size from 1 to 10 microns (easily visible with light microscopy).

### Demonstration of fluorogenic DNA assay

We show that the DNA immobilized on the surface can be designed to increase in fluorescence in response to an unlabeled oligonucleotide. We prepared a fluorogenic reporter complex for ssDNA C (see Supplemental Information for sequences). This was made of a final concentration of 1 µM TRC (HPLC purified), 1 µM Q_OSD_ (HPLC purified) in phosphate buffer. This was annealed as above. We incubated the particles (as generated above) with the DNA reporter complex. The particles were allowed to settle on a chamber slide and then incubated with various concentrations unlabeled ssDNA C (AnaC) oligonucleotide for 30 minutes. Samples of the particles before and after the addition of various concentrations of the ssDNA were observed on the fluorescent microscope (LumaScope 620, EtaLuma, Carlsbad, CA). This microscope uses 3 LED illumination sources to illuminate different fluorophores. False color images are generated by combining a red channel (λ_ex_ 580 nm), green channel (λ_ex_ 490 nm) and blue channel (λ_ex_ 400 nm).

### Generation of fluorescein- and Cascade Blue-labeled poly-T

Dual-modified acrydite/fluorescein poly-T DNA was acquired from IDT and used without further purification. Dual-modified 5′-acrydite and 3′-amine poly-T DNA was acquired from IDT and derivatized with Cascade Blue succinimidyl ester (Thermo-Fisher, Waltham, MA) according to manufacturer’s instructions. The resulting product was then purified using size exclusion columns (Thermo-Fisher, Waltham, MA). The product was stored at −20 °C in water until needed.

### Multiplexed red-fluorescent detection with blue/green fluorescence barcoding

Three different versions of the Texas Red DNA (denoted TRA, TRB and TRC) were designed with three corresponding oligonucleotides (denoted ssDNA A, ssDNA B, and ssDNA C). The designs differed only in their toehold regions and so shared a common quencher (denoted Q_OSD_). The three reporter complexes were immobilized onto three different particle batches as described above. In order to differentiate the particle types, the particles were generated with different ratios of fluorescently-labeled polyT DNA. Particles bearing TRA were dyed blue by including 2 μM pacific blue conjugated acrydite-DNA. Particles bearing TRB were dyed teal by including 6 μM fluorescein conjugated acrydite-DNA and 2 μM pacific blue conjugated acrydite-DNA. Particles bearing TRC were dyed green by including 1 μM fluorescein conjugated acrydite-DNA. These particles were added to a chamber slide and allowed to settle. The chamber was then gently rinsed with reaction buffer. Particles were then observed with a fluorescence microscope and their fluorescence quantified using automated image analysis software (see Supplemental Information). Data representing strong blue, green, or blue/green fluorescence were sorted into appropriate categories (Fig. 5(a)). The appropriate ssDNA was then added (ssDNA A, 1 μM; ssDNA B SNP, 6 μM; ssDNA C, 1 μM) and the samples were incubated for 15 minutes. The particles were again observed with a fluorescence microscope and their fluorescence quantified.

### PAGE analysis of catalytic assembly circuit

We ran native PAGE analysis of our adapted Zhang EDA circuit. We made a 10% polyacrylamide gel with sodium borate running buffer. We loaded 2 μl of NEB low molecular weight ladder in lanes 1 and 8. We assembled reactions and controls as follows: Lane 2, Negative Control: TR-SB-Q_EDA_ complex at 2 μM. Lane 3, Intermediate control: TR-SB-Q_EDA_ complex at 2 μM and mono-valent catalyst extended with 10 bases of poly-T at 2uM. Lane 4, No-catalyst (reaction leakage) control: TR-SB-Q_EDA_ complex at 2 μM and Fuel extended with 10 bases of poly T at 10 μM. Lane 5, Positive Control: TR at 2 μM and Fuel extended with 10 bases of poly T at 20 μM. Lane 6, Experimental: TR-SB-Q_EDA_ complex at 2 μM, catalyst at 2 uM and Fuel extended with 10 bases of poly T at 10 μM. Lane 7, Experimental with extended catalyst: TR-SB-Q_EDA_ complex at 2 μM, catalyst extended by 10 bases of poly T at 2 uM, and Fuel extended with 10 bases of poly T at 10 μM.

### Amplification of fluorescent signal using catalytic DNA circuit

DNA circuit components were assembled in circuit buffer (50 mM phosphate, 500 mM NaCl, pH 6.8). TR and a slight excess of Q were annealed. A slight excess of SB was added and the mixture was allowed to react for 30 min. The final concentration of the TR-SB-Q_EDA_ complex was 1000 nM and the final concentration of fuel was 1000 nM (in all cases except negative control from which fuel was omitted). A 384 well plate was blocked with superblock (Thermo-Fisher, Waltham, MA) for 1 hour at room temperature then washed with circuit buffer. Catalyst DNA/DNA walker or buffer was then added to the TR-SB-Q_EDA_ solution achieve the final concentration shown in Fig. 2(a). The samples were then transferred to the prepared 384 well plate. The plate was read with a fluorescence plate reader (Beckman-Coulter, Pasadena, CA).

### Detection of global DNA walker by particles

We prepared green particles bearing the TR-SB-Q_EDA_ DNA. The complex was assembled as described above. The particles were prepared with capture DNA on their surface also as described above. The TR-SB-Q_EDA_ complex was incubated with the particles for 1 hour then excess complex was washed away. The particles were then refrigerated until use. We suspended these particles in 18 μl of circuit buffer and allowed the particles to settle to the bottom of a chamber slide. We then added 1 μl of 5 μM fuel and 1 μl of the appropriate 20x concentration of DNA walker (to a final concentration shown in Fig. 2(c)). We acquired fluorescence data after incubating for 1 hour at 37 °C. The red fluorescence intensity was quantified using automated image analysis software (see Supplemental Information) and is shown in Fig. 2.

### Microscopically localized detection of diffusing DNA walker

Blue particles were prepared as above bearing the TR-Q_EDA_ complex; the SB was omitted and DNA walker was substituted. We denote these particles “release particles” as fuel can directly displace the DNA walker (see detailed schematic in Supplemental Information, Fig. SI3). We prepared green particles bearing the TR-SB-Q_EDA_ complex as “detector particles” for the DNA walker as described above. We mixed 1 μl of each particle type and 18 μl of circuit buffer and allowed the particles to settle to the bottom of a chamber slide. We then added 1 μl of 5 μM Fuel (but did not add additional DNA walker). We added more 20 μl circuit buffer before incubating. We acquired fluorescence data after incubating for 1 hour at 37 °C. The red fluorescence intensity of the green and blue particles was quantified using automated image analysis software (see Supplemental Information) and is shown in Fig. 2(b).

We tested the persistence of the non-walker catalyst and the walker catalyst on Green particles bearing the TR-SB-Q_EDA_ complex. The particles were prepared in the same way as detector particles as described above. 50 nM single-domain catalyst (non-walker) was added to 10 µl of particles in order to “prime” the particles with catalyst. The particles were washed three times with circuit buffer to remove excess, unbound catalyst. 1 µl of washed, primed particles were added to 18 µl of circuit buffer followed by 1 µl of 5 µM of fuel. Incubation was carried out at 37 °C for 1 hour. The results were viewed using an epifluorescence microscope. We added additional 100 nM of single catalyst in the positive control case. The equivalent experiment was for double catalyst with 25 nM priming catalyst (equivalent number of active domains). An additional 50 nM of double catalyst was added for the positive control case.

## Electronic supplementary material

Supplemental Information
