# Supplementary material for: Biomimetic Molecular Signaling using DNA Walkers on Microparticles
Source: Sci Rep. 2017 Jun 22;7:4081. doi: 10.1038/s41598-017-04316-1 (PMC5481426; doi:10.1038/s41598-017-04316-1)
Supplement: Supplementary file 1 — Supplemental Information [file 41598_2017_4316_MOESM1_ESM.pdf]

# Biomimetic molecular signaling using DNA Walkers on Microparticles

**Tulsi Ram Damase, Adam Spencer, Bamidele Samuel, Peter B. Allen***

## Supplemental Information Online:

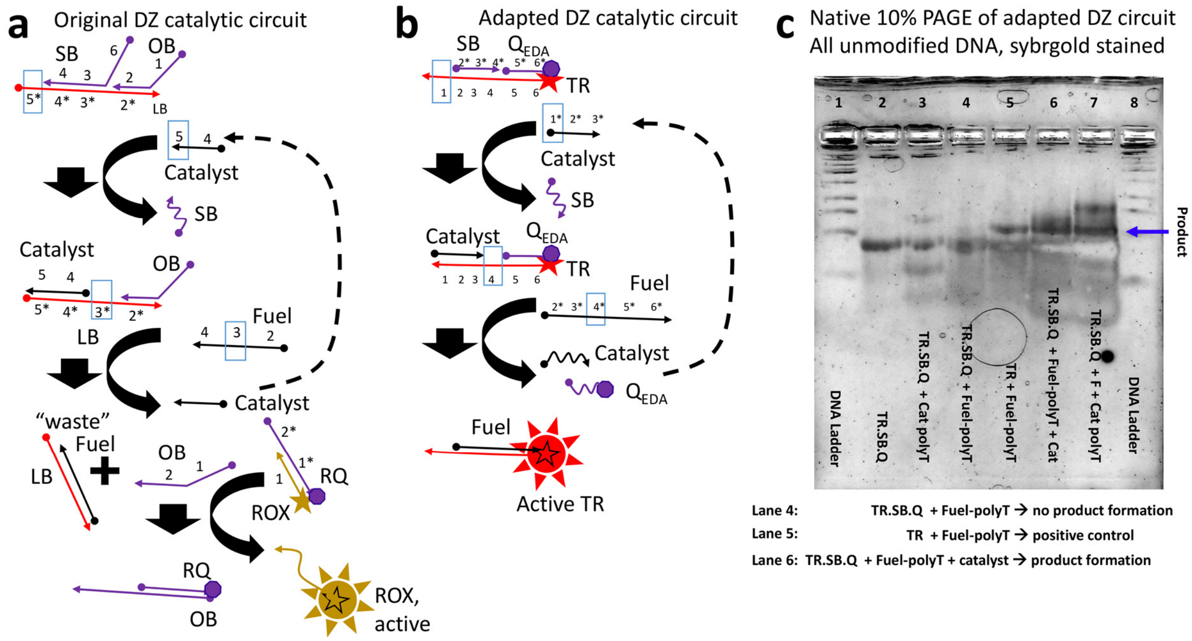


**Figure SI1:** (a) Schematic of the original EDA circuit by Zhang et. al.[21] reorganized to show the comparison to our adapted, fluorogenic reaction. Open toeholds are highlighted in blue boxes. (b) Detailed schematic our adapted reaction. (c) Native PAGE gel stained with SybrGold shows the operation of the adapted circuit.

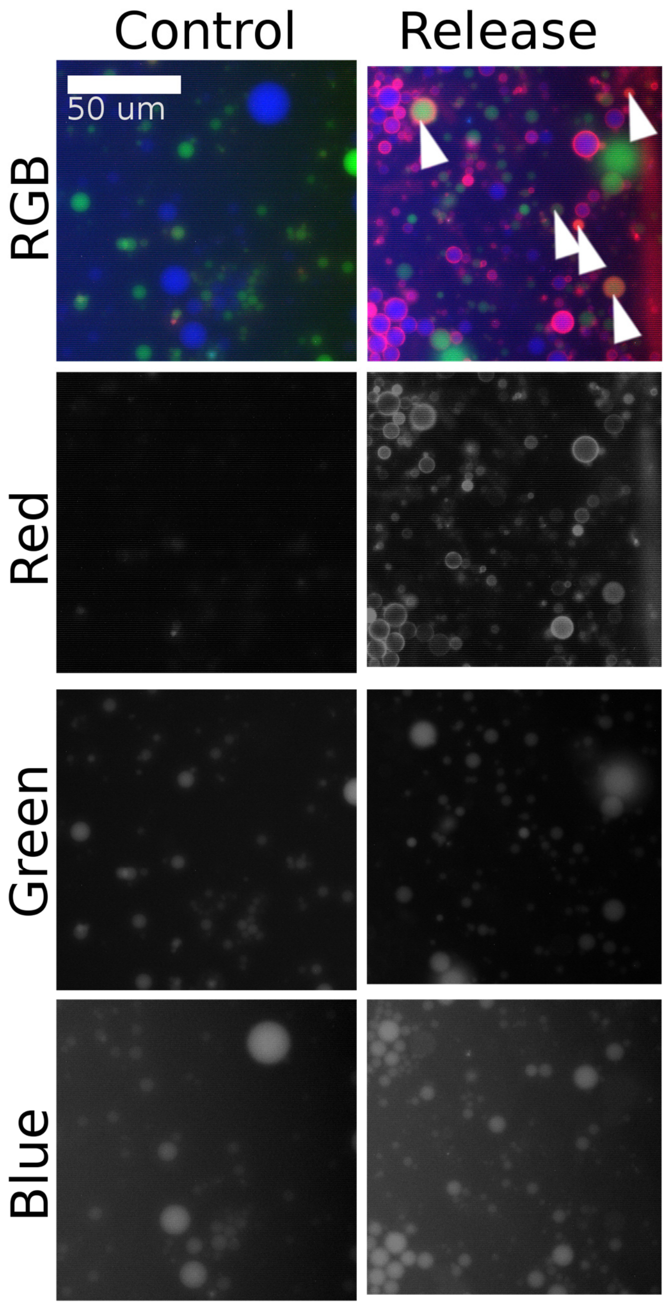


**Figure SI2:** Red, green, and blue channels of image data from Figure 1.

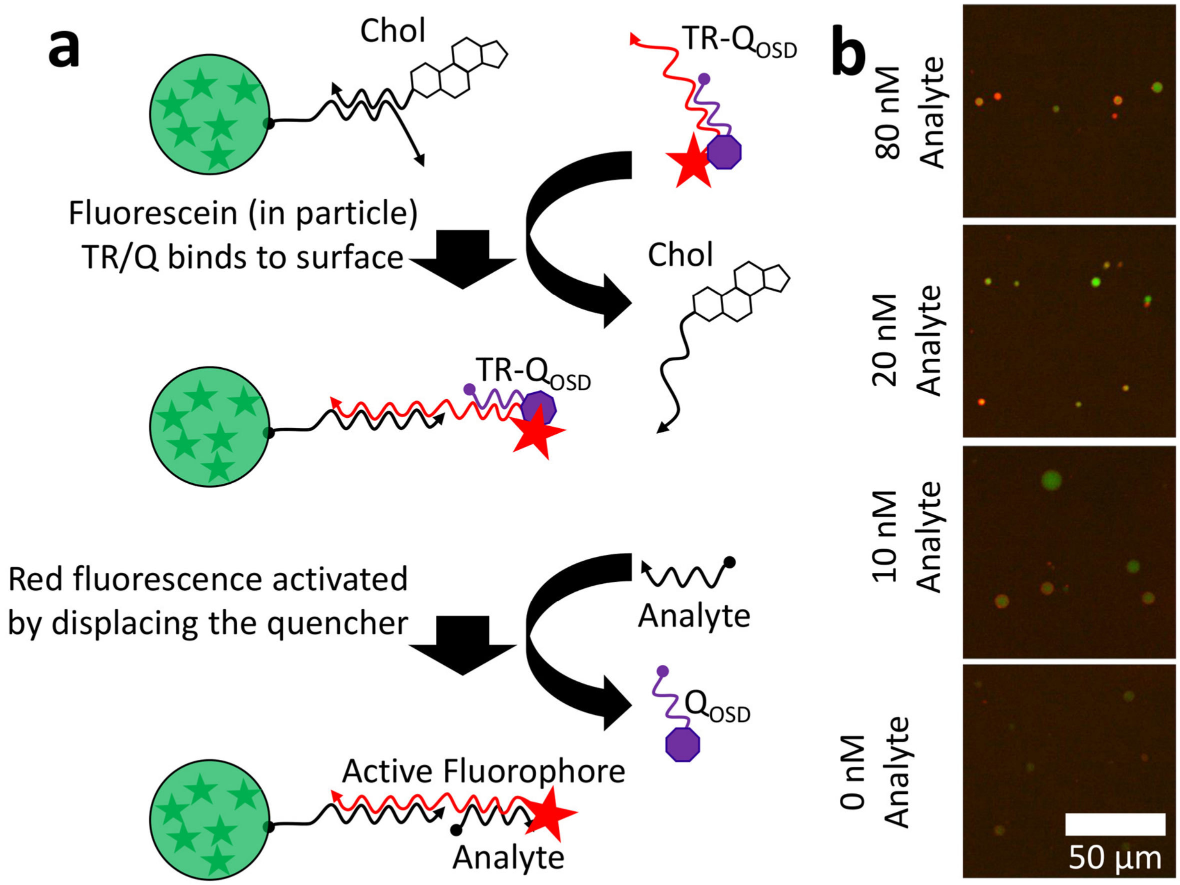


**Figure SI3:** We measured the limit of detection for the OSD reaction using a laser confocal microscope rather than a LED epifluorescence microscope. (a) A schematic shows the reaction where the fluorogenic complex displaces the cholesterol-modified DNA at the surface. The ssDNA then is able to displace the quencher and activate the Texas Red fluorophore. The particles contain fluorescein for identification. (b) The result showed fluorescence at 10 nM of the ssDNA.

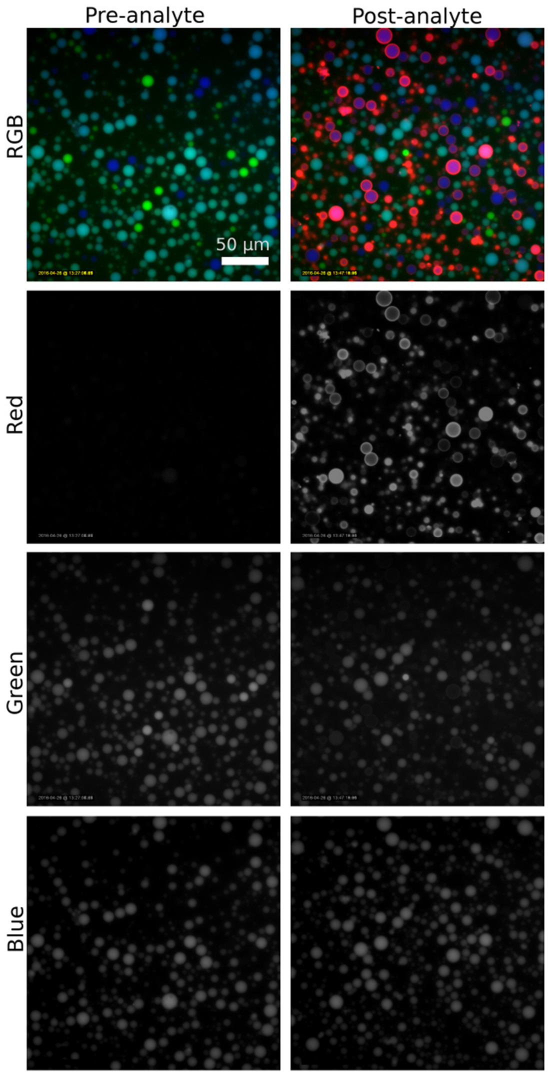


**Figure SI4:** Grayscale images corresponding to the microscopy data shown in Figure 4.

**Table S1:** Sequences for all DNA.

| | |
|---|---|
| <u>Multiplex OSD components</u> | |
| $Q_{OSD}$ | AATTCGGCCTGGAATA 3′Iowa black Red Quencher |
| Chol.A | GTCTCTGTGCCGCTATAATTTT 3′Cholesterol (HPLC) |
| Acryd.A | 5′Acrydite ATTATAGCGGCACAGAGACTAAGGTCGG (HPLC) |
| TRA | 5′Texas Red TATTCCAGGCCGAATT AACAAACT G CCGACCTTAGTCTCTGTGCCGCTATAAT (HPLC) |
| Chol.B | ATCAACTA CCTACTATTTT 3′Cholesterol (HPLC) |
| Acryd.B | 5′Acrydite ATAGTAGG TAGTTGAT ATTGGTAG (HPLC) |
| TRB | 5′Texas Red TATTCCAG GCCGAATT CACTCCGAG CTACCAAT ATCAACTA CCTACTAT |
| Chol.C | AACACTCA ACGCTACCTTT 3′Cholesterol (HPLC) |
| Acryd.C | 5′Acrydite GGTAGCGT TGAGTGTT AAAGTAGG (HPLC) |
| TRC | 5′Texas Red TATTCCAG GCCGAATT CTCTAAATG CCTACTTT AACACTCA ACGCTACC |
| Pacific blue Poly-T | 5′Acrydite TTTTTT 3′Pacific Blue |
| Fluorescein Poly-T | 5′Acrydite TTTTTT 3′Fluorescein |
| ssDNA A | 5′AGTTTGTT AATTCGGC CTGGAATA |
| ssDNA B | 5′TCGGAGTG AATTCGGC CTGGAATA |
| ssDNA B SNP (SNP underlined) | 5′TCGGAGT<u>C</u> AATTCGGC CTGGAATA |
| ssDNA C | 5′ATTTAGAG AATTCGGC CTGGAATA |
| | |
| <u>EDA circuit components</u> | |
| Br | 5′Texas Red TCTCCAACTAACTTACGG CCCT CATTCAATACCCTACG TCTCCA CCTACTTT AACACTCA ACGCTACC HPLC |
| $Q_{EDA}$ | CCGTAAGTTAGTTGGAGA 3′Iowa black Red Quencher |
| Cat | TGGAGACGTAGGGTATTGAATGAGAGTGGAGATGGGAGTAGTTGGAGACGTAGGGTATTG AATG |
| Fuel | CGTAGGGTATTGAATG AGGG CCGTAAGTTAGTTGGAGA |
| SB | CGTAGGGTATTGAATG AGGG |
| Cat-polyT | TGGAGA CGTAGGGTATTGAATG TTTTTTTTTTT |
| Fuel-polyT | CGTAGGGTATTGAATG AGGG CCGTAAGTTAGTTGGAGA TTTTTTTTTTT |

```
#Python Source Code
#Written in Anaconda, Python 2.7, iPython notebook
#Begin 160425 Local release from blue, detection by green - Fig1

# -*- coding: utf-8 -*-
# <nbformat>3.0</nbformat>

# <codecell>

#import packages to find the filenames and format the list
import numpy as np
import glob
#get a list of tiff files with green fluorescence data
numOfFiles=len(glob.glob('./160425_GreenCdetector*/*F1*'))
fileList=np.array([\
                   glob.glob('./160425_GreenCdetector*/*F3*'),\
                   glob.glob('./160425_GreenCdetector*/*F2*'),\
                   glob.glob('./160425_GreenCdetector*/*F1*'),\
                   np.zeros(numOfFiles),\
                   np.zeros(numOfFiles)])
#format the list and add a column of zeros to be replaced with average intensity value
fileList=np.transpose(fileList)
[fileList[0,0],fileList[0,1],fileList[0,2],fileList[0,3],fileList[0,4]]

# <codecell>

#PRELIMINARY LOADING OF ALL OF THE DEPENDENCIES
import skimage.io as ski
import scipy
import matplotlib.pyplot as plt
from skimage import morphology as mski
from skimage.morphology import disk
import glob
%matplotlib inline

#MAKE FUNCTIONS TO LOAD, BIN, BACKGROUND SUBTRACT THE IMAGE
def AVG_pixel(Im):
    row,col = np.shape(Im)
    x = np.floor((row-50)/2) #note that we cut off the bottom 50 pixels to remove time/date stamp
    y = np.floor(col/2)
    new_Image = np.zeros([int(x),int(y)],dtype=float)

    for i in range(1,int(x)):
        for j in range(1,int(y)):
            new_Image[i-1,j-1] = float(Im[2*i-1,2*j-1])+float(Im[2*i-2, 2*j-2])+float(Im[2*i-
1,2*j-2])+float(Im[2*i-2,2*j-1])
    return new_Image

def loadBinBGFile(fileName, color):
    I=ski.imread(fileName)      #read in a file
    I = AVG_pixel(I[:,:,color]) #bin the file using the above function
    A = np.size(I)
    Red_list = np.reshape(I,[1,A])
    #The channel intensities are sorted in an acending list
    Red_list = np.sort(Red_list)
    #The minimum background is removed from the image by taking the first 10% value
    min_back_red = Red_list[0,np.floor(0.1*A)]
    I = I-min_back_red
    I = np.clip(I,0.,Red_list[0,-1])
    return I

#process and clean up the mask to remove small and
#very large objects also smooth edges
def processMask(mask):
    mask = mski.remove_small_objects(mask, min_size=50)
    mask = mask-mski.remove_small_objects(mask, min_size=1900)
    mask = mski.opening(mask, disk(3))
    mask = mski.closing(mask, disk(3))
    mask = mski.dilation(mask,disk(3))
    return mask
```

```
#This function takes an image and a labeled mask (created later with scipy.ndimage.label)
#it resurns a list of the average intensity of each object in the mask
def measureRedObjectsFromMask(I2, LabelMask):
    results=np.zeros(LabelMask[1])
    maxDim=len(I2[0])-1
    for j in range(1,LabelMask[1]):
        currentObject=(LabelMask[0]==j)
        currentObjectPoints=np.nonzero(currentObject)

currentObjectPointsAdjusted=(np.clip(currentObjectPoints[0],0,maxDim),np.clip(currentObjectPoints
[1],0,maxDim))
        results[j]=np.mean(I2[currentObjectPointsAdjusted])
    return results

# <codecell>

#LOAD THE INITIAL GREEN/BLUE IMAGE AND VISUALLY SHOW HOW THE MASK WILL BE MADE
i=1
IR=loadBinBGFile(fileList[i,0], 0)
IG=loadBinBGFile(fileList[i,1], 1)
IB=loadBinBGFile(fileList[i,2], 2)
#MAKE THE MASK
#A mask is created by thresholding the image, all pixels above the value specified
# are set as 1 while those bellow are 0
#thresholds are set heuristically based on inspection;
#thresholds are used consistently for the remaining files
gthresh=30
bthresh=240
mask = np.logical_or((IG > gthresh),(IB > bthresh))
tealmask= np.logical_and((IG > gthresh),(IB > bthresh))
greenmask = mask-(IB > bthresh)
bluemask =  mask-(IG > gthresh)

#the masks are labeled so that each disconencted object is set to a unique value
tealmask=processMask(tealmask)
Labeltealmask=scipy.ndimage.label(tealmask)
greenmask=processMask(greenmask)
Labelgreenmask=scipy.ndimage.label(greenmask)
bluemask=processMask(bluemask)
Labelbluemask=scipy.ndimage.label(bluemask)

#SHOW THE RESULTS
fig, axes = plt.subplots(ncols=3, figsize=(10, 5), sharex=True, sharey=True,
subplot_kw={'adjustable':'box-forced'})
axes[0].imshow(IB+IG, cmap=plt.cm.gray)
axes[0].set_title('Image')

axes[1].imshow((greenmask > 0), cmap=plt.cm.gray)
axes[1].set_title('maskG')

axes[2].imshow((bluemask > 0), cmap=plt.cm.gray)
axes[2].set_title('maskB')

for ax in axes:
    ax.axis('off')
plt.show()

# <codecell>

#DEMONSTRATION OF HOW THE MASKS CAN BE MEASURED TO PRODUCE THE SCATTER PLOT
#This shows how the blue (release) and green (detectors) particles are proccessed separately.
#There are no teal particles; objects that are overlapping are discarded implicitly.

results=np.zeros([Labelbluemask[1]+Labelgreenmask[1],2])

#
i=0
maxDim=len(IR[0])-1
for j in range(1,Labelbluemask[1]):
    currentObject=(Labelbluemask[0]==j)
    currentObjectPoints=np.nonzero(currentObject)
```

```
currentObjectPointsAdjusted=(np.clip(currentObjectPoints[0],0,maxDim),np.clip(currentObjectPoints
[1],0,maxDim))
    results[i,0]=np.mean(IG[currentObjectPointsAdjusted])
    results[i,1]=np.mean(IB[currentObjectPointsAdjusted])
    i=i+1
for j in range(1,Labelgreenmask[1]):
    currentObject=(Labelgreenmask[0]==j)
    currentObjectPoints=np.nonzero(currentObject)

currentObjectPointsAdjusted=(np.clip(currentObjectPoints[0],0,maxDim),np.clip(currentObjectPoints
[1],0,maxDim))
    results[i,0]=np.mean(IG[currentObjectPointsAdjusted])
    results[i,1]=np.mean(IB[currentObjectPointsAdjusted])
    i=i+1


plt.scatter(results[:,0],results[:,1])

# <codecell>

redobjects=measureRedObjectsFromMask(IR,Labelgreenmask)
plt.hist(redobjects)
redobjects=measureRedObjectsFromMask(IR,Labelbluemask)
plt.hist(redobjects)

# <codecell>

#DEMONSTRATION OF HOW THE MASK IS USED TO PROCESS A RED IMAGE
for i in range(len(fileList[:,0])):
    #LOAD THE IMAGES
    IR=loadBinBGFile(fileList[i,0], 0)
    IG=loadBinBGFile(fileList[i,1], 1)
    IB=loadBinBGFile(fileList[i,2], 2)
    #MAKE THE MASK
    #A mask is created by thresholding the image, all pixels above the value specified
    # are set as 1 while those bellow are 0
    gthresh=30
    bthresh=240
    mask = np.logical_or((IG > gthresh),(IB > bthresh))
    tealmask= np.logical_and((IG > gthresh),(IB > bthresh))
    greenmask = mask-(IB > bthresh)
    bluemask =  mask-(IG > gthresh)
    tealmask=processMask(tealmask)
    Labeltealmask=scipy.ndimage.label(tealmask)
    greenmask=processMask(greenmask)
    Labelgreenmask=scipy.ndimage.label(greenmask)
    bluemask=processMask(bluemask)
    Labelbluemask=scipy.ndimage.label(bluemask)


    fileList[i,3]=np.average(measureRedObjectsFromMask(IR,Labelgreenmask))
    fileList[i,4]=np.average(measureRedObjectsFromMask(IR,Labelbluemask))
    print(fileList[i,:])


# <codecell>

np.savetxt("160425_release_detect_summary.csv", fileList, fmt="%s", delimiter=",")

# <codecell>

fileList
```

```
#begin 160424 Amplifier circuit on surface green particles - Fig2
# -*- coding: utf-8 -*-
# <nbformat>3.0</nbformat>

# <codecell>

#import packages to find the filenames and format the list
import glob
import numpy as np
#get a list of tiff files with green fluorescence data
fileListG=glob.glob('./160424*/*F2*')
#get a list of tiff files with red fluorescence data
fileListR=glob.glob('./160424*/*F3*')
#format the list and add a column of zeros to be replaced with average intensity value
fileList=np.transpose(np.array([fileListG,fileListR,np.zeros(len(fileListG))]))

# <codecell>

#PRELIMINARY LOADING OF ALL OF THE DEPENDENCIES
import skimage.io as ski
import scipy
import matplotlib.pyplot as plt
from skimage import morphology as mski
from skimage.morphology import disk
import glob
%matplotlib inline

#MAKE A FUNCTION TO LOAD, BIN, BACKGROUND SUBTRACT THE IMAGE
def AVG_pixel(Im): #function bins an image by summing every 2x2 pixel block
    row,col = np.shape(Im)
    x = np.floor((row-50)/2) #not that we cut off the bottom 50 pixels to remove time/date stamp
    y = np.floor(col/2)
    new_Image = np.zeros([int(x),int(y)],dtype=float)

    for i in range(1,int(x)):
        for j in range(1,int(y)):
            new_Image[i-1,j-1] = float(Im[2*i-1,2*j-1])+float(Im[2*i-2, 2*j-2])+float(Im[2*i-
1,2*j-2])+float(Im[2*i-2,2*j-1])
    return new_Image

def loadBinBGFile(fileName, color):
    I=ski.imread(fileName) #read in a file
    I = AVG_pixel(I[:,:,color]) #bin the file using the above function
    A = np.size(I)
    Red_list = np.reshape(I,[1,A])
    #The channel intensities are sorted in an acending list
    Red_list = np.sort(Red_list)
    #The background is subtracted by calculating the 10th percentile intensity
    min_back_red = Red_list[0,np.floor(0.1*A)]
    I = I-min_back_red
    I = np.clip(I,0.,Red_list[0,-1])
    return I

# <codecell>

#DEMONSTRATE HOW A MASK IS CONSTRUCTED FROM THE GREEN IMAGE

#Load the initial green image
I=loadBinBGFile(fileList[2,0], 1)
#MAKE THE MASK
# A mask is created by thresholding the green image.
# All pixels above the value specified
# are set as 1 while those bellow are 0
mask = (I > 25) #threshold is set heuristically at 25 based on inspection;
                #threshold is used consistently for the remaining files
#the mask is refined by removing holes, small objects (20 pixels or less) and smoothed
mask = mski.remove_small_objects(mask, min_size=20)
mask = mask-mski.remove_small_objects(mask, min_size=1900)
mask = mski.opening(mask, disk(3))
mask = mski.closing(mask, disk(3))
```

```
#the mask is then labeled so that each disconenected object is set to a unique value
LabelMask=scipy.ndimage.label(mask)

# SHOW THE RESULTS
fig, axes = plt.subplots(ncols=2, figsize=(10, 5), sharex=True, sharey=True,
subplot_kw={'adjustable':'box-forced'})
axes[0].imshow(I, cmap=plt.cm.gray)
axes[0].set_title('Image')

axes[1].imshow(mask, cmap=plt.cm.gray)
axes[1].set_title('mask')

for ax in axes:
    ax.axis('off')
plt.show()

# <codecell>

#DEMONSTRATION OF HOW THE MASK IS USED TO PROCESS A RED IMAGE

#load the red image that corresponds to the mask
I2=loadBinBGFile(fileList[0,1], 0)
#make an array with the proper length to hold the average intensity of each object
results=np.zeros(LabelMask[1])


t0, t1 = (0,0)
maxDim=len(I2[0])-1
#for each object in the mask
for j in range(1,LabelMask[1]):
    currentObject=(LabelMask[0]==j) #get the pixels that correspond to that object
    currentObjectPoints=np.nonzero(currentObject) #get a list of the pixel coordinates
    #get a list of the pixel intensities for all of the pixels in that object

currentObjectPointsAdjusted=(np.clip(currentObjectPoints[0]+t0,0,maxDim),np.clip(currentObjectPoi
nts[1]+t1,0,maxDim))
    results[j]=np.mean(I2[currentObjectPointsAdjusted]) #average those pixel intensities and add
to the array

results #show the array for the sample image

# <codecell>

#REPEAT THE ABOVE DEMONSTRATION FOR EACH FILE IN THE LIST
for i in range(len(fileList[:,0])):
    #LOAD THE INITIAL GREEN IMAGE AND VISUALLY SHOW HOW THE MASK WILL BE MADE
    I=loadBinBGFile(fileList[i,0], 1)
    #MAKE THE MASK
    #A mask is created by thresholding the image, all pixels above the value specified
    # are set as 1 while those bellow are 0
    mask = (I > 25)
    mask = mski.remove_small_objects(mask, min_size=20)
    mask = mask-mski.remove_small_objects(mask, min_size=1900)
    mask = mski.opening(mask, disk(3))
    mask = mski.closing(mask, disk(3))
    LabelMask=scipy.ndimage.label(mask)

    I2=loadBinBGFile(fileList[i,1], 0)
    #make an array with the proper length to hold the average intensity of each object
    results=np.zeros(LabelMask[1])

    t0, t1 = (0,0)
    maxDim=len(I2[0])-1
    #for each object in the mask
    for j in range(1,LabelMask[1]):
        currentObject=(LabelMask[0]==j)
        currentObjectPoints=np.nonzero(currentObject)

currentObjectPointsAdjusted=(np.clip(currentObjectPoints[0]+t0,0,maxDim),np.clip(currentObjectPoi
nts[1]+t1,0,maxDim))
        results[j]=np.mean(I2[currentObjectPointsAdjusted])
```

```
    fileList[i,2]=np.mean(results)
    print(fileList[i,1], fileList[i,2])


# <codecell>

#save the array of averages to a text file with comma separated values
np.savetxt("160424_DZ_summary.csv", fileList, fmt="%s", delimiter=",")
```

```
# 160427 Green particles OSD Reaction LOD calculation - Fig3
# -*- coding: utf-8 -*-
# <nbformat>3.0</nbformat>

# <codecell>

#import packages to find the filenames and format the list
import numpy as np
import glob
#get a list of tiff files with red (F3), green (F2), and blue (F1) fluorescence data
fileListR=glob.glob('./160427_Multiplex_GreenC*/*F3*')
fileListG=glob.glob('./160427_Multiplex_GreenC*/*F2*')
fileListB=glob.glob('./160427_Multiplex_GreenC*/*F1*')
#format the list and add columns of zeros to be replaced with average intensity value
fileList=np.array([fileListR,fileListG,fileListB,np.zeros(len(fileListR)),np.zeros(len(fileListR)
),np.zeros(len(fileListR))])
fileList=np.transpose(fileList)
[fileList[0,:]]

# <codecell>

#PRELIMINARY LOADING OF ALL OF THE DEPENDENCIES
import skimage.io as ski
import scipy
import matplotlib.pyplot as plt
from skimage import morphology as mski
from skimage.morphology import disk
import glob
%matplotlib inline

#MAKE A FUNCTION TO BIN AND BACKGROUND SUBTRACT THE IMAGE
def AVG_pixel(Im): #function bins an image by summing every 2x2 pixel block
    row,col = np.shape(Im)
    x = np.floor((row-50)/2) #note that we cut off the bottom 50 pixels to remove time/date stamp
    y = np.floor(col/2)
    new_Image = np.zeros([int(x),int(y)],dtype=float)

    for i in range(1,int(x)):
        for j in range(1,int(y)):
            new_Image[i-1,j-1] = float(Im[2*i-1,2*j-1])+float(Im[2*i-2, 2*j-2])+float(Im[2*i-
1,2*j-2])+float(Im[2*i-2,2*j-1])
    return new_Image

#Read and bin an image
def loadBinBGFile(fileName, color):
    I=ski.imread(fileName)      #read in a file
    I = AVG_pixel(I[:,:,color]) #bin the file using the above function
    A = np.size(I)
    Red_list = np.reshape(I,[1,A])
    #The channel intensities are sorted in an acending list
    Red_list = np.sort(Red_list)
    #The minimum background is removed from the image by taking the first 10% value
    min_back_red = Red_list[0,np.floor(0.1*A)]
    I = I-min_back_red
    I = np.clip(I,0.,Red_list[0,-1])
    return I

#this function processes a mask by removing holes, small
#objects (50 pixels or less) and smoothing the edges
def processMask(mask):
    mask = mski.remove_small_objects(mask, min_size=50)
    mask = mski.opening(mask, disk(3))
    mask = mski.closing(mask, disk(3))
    mask = mski.dilation(mask,disk(3))
    return mask

#This function takes an image and a labeled mask (created later with scipy.ndimage.label)
#it resurns a list of the average intensity of each object in the mask
def measureRedObjectsFromMask(I2, LabelMask):
    results=np.zeros(LabelMask[1])
    maxDim=len(I2[0])-1
```

```
    for j in range(1,LabelMask[1]):
        currentObject=(LabelMask[0]==j)
        currentObjectPoints=np.nonzero(currentObject)

currentObjectPointsAdjusted=(np.clip(currentObjectPoints[0],0,maxDim),np.clip(currentObjectPoints
[1],0,maxDim))
        results[j]=np.mean(I2[currentObjectPointsAdjusted])
    return results

# <codecell>

#LOAD THE INITIAL GREEN IMAGE AND VISUALLY SHOW HOW THE MASK WILL BE MADE
IB=loadBinBGFile(fileList[0,2], 2)
IG=loadBinBGFile(fileList[0,1], 1)
IR=loadBinBGFile(fileList[0,0], 0)
#MAKE THE MASK
#A mask is created by thresholding the image, all pixels above the value specified
# are set as 1 while those bellow are 0
gthresh=40
bthresh=150
mask = np.logical_or((IG > gthresh),(IB > bthresh))
tealmask= processMask(np.logical_and((IG > gthresh),(IB > bthresh)))
greenmask = processMask(mask-(IB > bthresh))
bluemask =  processMask(mask-(IG > gthresh))

#SHOW THE RESULTS
fig, axes = plt.subplots(ncols=5, figsize=(20, 10), sharex=True, sharey=True,
subplot_kw={'adjustable':'box-forced'})
axes[0].imshow(IB, cmap=plt.cm.gray)
axes[0].set_title('BImage')

axes[1].imshow(IG, cmap=plt.cm.gray)
axes[1].set_title('Gimage')

axes[2].imshow(tealmask, cmap=plt.cm.gray)
axes[2].set_title('Tmask')

axes[3].imshow(greenmask, cmap=plt.cm.gray)
axes[3].set_title('Gmask')

axes[4].imshow(bluemask, cmap=plt.cm.gray)
axes[4].set_title('Bmask')

for ax in axes:
    ax.axis('off')
plt.show()

# <codecell>

#DEMONSTRATE THE PROCESSING OF A SINGLE IMAGE (fileList[1])
for i in range(1): #set file 1
    # LOAD THE INITIAL GREEN IMAGE AND VISUALLY SHOW HOW THE MASK WILL BE MADE
    IB=loadBinBGFile(fileList[i,2], 2)
    IG=loadBinBGFile(fileList[i,1], 1)
    # MAKE THE MASK
    # A mask is created by thresholding the image,
    # all pixels above the value specified
    # are set as 1 while those bellow are 0
    gthresh=40   #thresholds are set heuristically based on inspection;
    bthresh=150  #thresholds are used consistently for the remaining files
    mask = np.logical_or((IG > gthresh),(IB > bthresh))
    tealmask= np.logical_and((IG > gthresh),(IB > bthresh))
    greenmask = mask-(IB > bthresh)
    bluemask =  mask-(IG > gthresh)
    tealmask=scipy.ndimage.label(processMask(tealmask))
    greenmask=scipy.ndimage.label(processMask(greenmask))
    bluemask=scipy.ndimage.label(processMask(bluemask))

    I2=loadBinBGFile(fileList[i,0], 0)
    fileList[i,3]=np.average(measureRedObjectsFromMask(I2,tealmask))
    fileList[i,4]=np.average(measureRedObjectsFromMask(I2,greenmask))
```

```
    fileList[i,5]=np.average(measureRedObjectsFromMask(I2,bluemask))
    print(fileList[i,:])

# <codecell>

#PROCESS ALL IAMGES (fileList[all])
for i in range(len(fileList[:,0])):
    #LOAD THE INITIAL GREEN IMAGE AND VISUALLY SHOW HOW THE MASK WILL BE MADE
    IB=loadBinBGFile(fileList[i,2], 2)
    IG=loadBinBGFile(fileList[i,1], 1)
    #MAKE THE MASK
    #A mask is created by thresholding the image, all pixels above the value specified
    # are set as 1 while those bellow are 0
    gthresh=40
    bthresh=150
    mask = np.logical_or((IG > gthresh),(IB > bthresh))
    tealmask= np.logical_and((IG > gthresh),(IB > bthresh))
    greenmask = mask-(IB > bthresh)
    bluemask =  mask-(IG > gthresh)
    tealmask=scipy.ndimage.label(processMask(tealmask))
    greenmask=scipy.ndimage.label(processMask(greenmask))
    bluemask=scipy.ndimage.label(processMask(bluemask))

    I2=loadBinBGFile(fileList[i,0], 0)
    fileList[i,3]=np.percentile(measureRedObjectsFromMask(I2,tealmask),80)
    fileList[i,4]=np.percentile(measureRedObjectsFromMask(I2,greenmask),80)
    fileList[i,5]=np.percentile(measureRedObjectsFromMask(I2,bluemask),80)
    print(fileList[i,:])

# <codecell>

np.savetxt("160427_green_LOD_top50.csv", fileList, fmt="%s", delimiter=",")
```

```
#Begin 160426 Multiplex OSD reaction, scatter plot and orthogonality - Fig5
# -*- coding: utf-8 -*-
# <nbformat>3.0</nbformat>

# <codecell>

#import packages to find the filenames and format the list
import numpy as np
import glob
#get a list of tiff files with green fluorescence data
fileListR=glob.glob('./160426_Multiplex*/*F3*')
fileListG=glob.glob('./160426_Multiplex*/*F2*')
fileListB=glob.glob('./160426_Multiplex*/*F1*')
#get a list of tiff files with green fluorescence data
fileList=np.array([fileListR,fileListG,fileListB,np.zeros(len(fileListR)),np.zeros(len(fileListR)
),np.zeros(len(fileListR))])
fileList=np.transpose(fileList)
[fileList[0,:]]

# <codecell>

#PRELIMINARY LOADING OF ALL OF THE DEPENDENCIES
import skimage.io as ski
import scipy
import matplotlib.pyplot as plt
from skimage import morphology as mski
from skimage.morphology import disk
import glob
%matplotlib inline

#MAKE A FUNCTION TO LOAD, BIN, BACKGROUND SUBTRACT THE IMAGE
def AVG_pixel(Im):
    row,col = np.shape(Im)
    x = np.floor((row-50)/2) #note that we cut off the bottom 50 pixels to remove time/date stamp
    y = np.floor(col/2)
    new_Image = np.zeros([int(x),int(y)],dtype=float)

    for i in range(1,int(x)):
        for j in range(1,int(y)):
            new_Image[i-1,j-1] = float(Im[2*i-1,2*j-1])+float(Im[2*i-2, 2*j-2])+float(Im[2*i-
1,2*j-2])+float(Im[2*i-2,2*j-1])
    return new_Image

def loadBinBGFile(fileName, color):
    I=ski.imread(fileName)
    I = AVG_pixel(I[:,:,color])
    A = np.size(I)
    Red_list = np.reshape(I,[1,A])
    #The channel intensities are sorted in an acending list
    Red_list = np.sort(Red_list)
    #The minimum background is removed from the image by taking the first 10% value
    min_back_red = Red_list[0,np.floor(0.1*A)]
    I = I-min_back_red
    I = np.clip(I,0.,Red_list[0,-1])
    return I

def processMask(mask):
    mask = mski.remove_small_objects(mask, min_size=50)
    mask = mask-mski.remove_small_objects(mask, min_size=1900)
    mask = mski.opening(mask, disk(3))
    mask = mski.closing(mask, disk(3))
    mask = mski.dilation(mask,disk(3))
    return mask

def measureRedObjectsFromMask(I2, LabelMask):
    results=np.zeros(LabelMask[1])
    maxDim=len(I2[0])-1
    for j in range(1,LabelMask[1]):
        currentObject=(LabelMask[0]==j)
        currentObjectPoints=np.nonzero(currentObject)
```

```
currentObjectPointsAdjusted=(np.clip(currentObjectPoints[0],0,maxDim),np.clip(currentObjectPoints
[1],0,maxDim))
        results[j]=np.mean(I2[currentObjectPointsAdjusted])
    return results

# <codecell>

#DEMONSTRATE HOW A MASK IS CONSTRUCTED FROM THE BLUE, GREEN AND TEAL
IB=loadBinBGFile(fileList[0,2], 2)
IG=loadBinBGFile(fileList[0,1], 1)
#MAKE THE MASK
#A mask is created by thresholding the image, all pixels above the value specified
#are set as 1 while those bellow are 0
#thresholds are set heuristically based on inspection;
#thresholds are used consistently for the remaining files
gthresh=40
bthresh=150
mask = np.logical_or((IG > gthresh),(IB > bthresh))
tealmask= processMask(np.logical_and((IG > gthresh),(IB > bthresh)))
greenmask = processMask(mask-(IB > bthresh))
bluemask =  processMask(mask-(IG > gthresh))

#SHOW THE RESULTS
fig, axes = plt.subplots(ncols=5, figsize=(20, 10), sharex=True, sharey=True,
subplot_kw={'adjustable':'box-forced'})
axes[0].imshow(IB, cmap=plt.cm.gray)
axes[0].set_title('BImage')

axes[1].imshow(IG, cmap=plt.cm.gray)
axes[1].set_title('Gimage')

axes[2].imshow(tealmask, cmap=plt.cm.gray)
axes[2].set_title('Tmask')

axes[3].imshow(greenmask, cmap=plt.cm.gray)
axes[3].set_title('Gmask')

axes[4].imshow(bluemask, cmap=plt.cm.gray)
axes[4].set_title('Bmask')

for ax in axes:
    ax.axis('off')
plt.show()

# <codecell>

#PROCESS THE MASKS IDENTIFIED ABOVE AND
labeltealmask=scipy.ndimage.label(tealmask)
labelgreenmask=scipy.ndimage.label(greenmask)
labelbluemask=scipy.ndimage.label(bluemask)
results=np.zeros([labelbluemask[1]+labelgreenmask[1]+labeltealmask[1],2])

#For each object in the complete list, make an entry in the list of the results
#with the blue and green average intensity for that object to set up the scatterplot.
i=0
maxDim=len(IB[0])-1
for j in range(1,labelbluemask[1]):
    currentObject=(labelbluemask[0]==j)
    currentObjectPoints=np.nonzero(currentObject)

currentObjectPointsAdjusted=(np.clip(currentObjectPoints[0],0,maxDim),np.clip(currentObjectPoints
[1],0,maxDim))
    results[i,0]=np.mean(IG[currentObjectPointsAdjusted])
    results[i,1]=np.mean(IB[currentObjectPointsAdjusted])
    i=i+1
for j in range(1,labelgreenmask[1]):
    currentObject=(labelgreenmask[0]==j)
    currentObjectPoints=np.nonzero(currentObject)
```

```
currentObjectPointsAdjusted=(np.clip(currentObjectPoints[0],0,maxDim),np.clip(currentObjectPoints
[1],0,maxDim))
    results[i,0]=np.mean(IG[currentObjectPointsAdjusted])
    results[i,1]=np.mean(IB[currentObjectPointsAdjusted])
    i=i+1
for j in range(1,labeltealmask[1]):
    currentObject=(labeltealmask[0]==j)
    currentObjectPoints=np.nonzero(currentObject)

currentObjectPointsAdjusted=(np.clip(currentObjectPoints[0],0,maxDim),np.clip(currentObjectPoints
[1],0,maxDim))
    results[i,0]=np.mean(IG[currentObjectPointsAdjusted])
    results[i,1]=np.mean(IB[currentObjectPointsAdjusted])
    i=i+1

plt.figure(num=None, figsize=(12, 7), dpi=80, facecolor='w', edgecolor='k')
plt.scatter(results[:,0],results[:,1])

# <codecell>

for i in range(len(fileList[:,0])):
    #LOAD THE INITIAL GREEN IMAGE AND VISUALLY SHOW HOW THE MASK WILL BE MADE
    IB=loadBinBGFile(fileList[i,2], 2)
    IG=loadBinBGFile(fileList[i,1], 1)
    #MAKE THE MASK
    #A mask is created by thresholding the image, all pixels above the value specified
    # are set as 1 while those bellow are 0
    gthresh=40
    bthresh=150
    mask = np.logical_or((IG > gthresh),(IB > bthresh))
    tealmask= np.logical_and((IG > gthresh),(IB > bthresh))
    greenmask = mask-(IB > bthresh)
    bluemask =  mask-(IG > gthresh)
    tealmask=scipy.ndimage.label(processMask(tealmask))
    greenmask=scipy.ndimage.label(processMask(greenmask))
    bluemask=scipy.ndimage.label(processMask(bluemask))

    I2=loadBinBGFile(fileList[i,0], 0)
    fileList[i,3]=np.average(measureRedObjectsFromMask(I2,tealmask))
    fileList[i,4]=np.average(measureRedObjectsFromMask(I2,greenmask))
    fileList[i,5]=np.average(measureRedObjectsFromMask(I2,bluemask))
    print(fileList[i,:])

# <codecell>

np.savetxt("160426_multiplex_redo.csv", fileList, fmt="%s", delimiter=",")
```
